# Formation of Common Mycorrhizal Networks Significantly Affects Plant Biomass and Soil Properties of the Neighboring Plants under Various Nitrogen Levels

**DOI:** 10.3390/microorganisms8020230

**Published:** 2020-02-08

**Authors:** Muhammad Atif Muneer, Ping Wang, Jing Zhang, Yaoming Li, Muhammad Zeeshan Munir, Baoming Ji

**Affiliations:** 1 School of Grassland Science, Beijing Forestry University, Beijing 100083, China; m_atifmuneer@yahoo.com (M.A.M.); wangping@bjfu.edu.cn (P.W.); yaomingli@bjfu.edu.cn (Y.L.); 2 School of Biological Sciences and Technology, Beijing Forestry University, Beijing 100083, China; zeeshanmunir1270@gmail.com

**Keywords:** mycorrhizal network, nitrogen addition, plant growth, soil properties, ^15^N transfer, *Cleistogene squarrosa*, Inner Mongolia

## Abstract

Common mycorrhizal networks (CMNs) allow the transfer of nutrients between plants, influencing the growth of the neighboring plants and soil properties. *Cleistogene squarrosa* (*C. squarrosa*) is one of the most common grass species in the steppe ecosystem of Inner Mongolia, where nitrogen (N) is often a key limiting nutrient for plant growth, but little is known about whether CMNs exist between neighboring individuals of *C. squarrosa* or play any roles in the N acquisition of the *C. squarrosa* population. In this study, two *C. squarrosa* individuals, one as a donor plant and the other as a recipient plant, were planted in separate compartments in a partitioned root-box. Adjacent compartments were separated by 37 µm nylon mesh, in which mycorrhizal hyphae can go through but not roots. The donor plant was inoculated with arbuscular mycorrhizal (AM) fungi, and their hyphae potentially passed through nylon mesh to colonize the roots of the recipient plant, resulting in the establishment of CMNs. The formation of CMNs was verified by microscopic examination and ^15^N tracer techniques. Moreover, different levels of N fertilization (N0 = 0, N1 = 7.06, N2 = 14.15, N3 = 21.19 mg/kg) were applied to evaluate the CMNs’ functioning under different soil nutrient conditions. Our results showed that when *C. squarrosa–C. squarrosa* was the association, the extraradical mycelium transferred the ^15^N in the range of 45–55% at different N levels. Moreover, AM fungal colonization of the recipient plant by the extraradical hyphae from the donor plant significantly increased the plant biomass and the chlorophyll content in the recipient plant. The extraradical hyphae released the highest content of glomalin-related soil protein into the rhizosphere upon N2 treatment, and a significant positive correlation was found between hyphal length and glomalin-related soil proteins (GRSPs). GRSPs and soil organic carbon (SOC) were significantly correlated with mean weight diameter (MWD) and helped in the aggregation of soil particles, resulting in improved soil structure. In short, the formation of CMNs in this root-box experiment supposes the existence of CMNs in the typical steppe plants, and CMNs-mediated N transfer and root colonization increased the plant growth and soil properties of the recipient plant.

## 1. Introduction

Arbuscular mycorrhizal (AM) symbiosis between higher plant roots and the fungi belonging to the phylum Glomeromycota is one of the most common mutualistic associations in terrestrial ecosystems [1,2]. In AM symbionts, the fungi act as an interface between plant roots and soil, thereby helping the host plant in the acquisition of limiting soil nutrients, such as phosphorus and nitrogen (N). One key characteristic feature of AM fungi is that their hyphae can penetrate into root cortical cells to form intraradical structures and extend outside the roots to form extraradical hyphae in the rhizosphere [3]. Moreover, extensively branched extraradical mycelia can interconnect neighboring plants to form common mycorrhizal networks (CMNs) [4,5,6]. These CMNs can affect the distribution of mineral nutrients like carbon [7,8], N [9], and phosphorus [10] among the connected plants. This could ultimately influence the plant’s establishment [11,12], survival [13,14], growth [15] and physiology [16,17]. However, the underground network is very complex, and a deep understanding of CMN’s formation, existence and functioning requires microscopic or tracer element techniques. The application of an N stable isotope tracer technique has confirmed the transfer of nutrients between CMN-connected plants. For example, a CMN was established by the native AM fungi between the grasses (*Nassella pulchra*, *Bromus madritensis*, and *B. hordeaceus*) and the forbs (*Trifolium microcephalum*, *Sanicula bipinnata*, and *Madia gracilis*) and CMNs exhibited N communication between the plants [18]. Barto et al. (2011) also studied the transfer of allelochemicals from source plant to target plant of *Tagetes tenuifolia* with the help of CMNs [19]. It is also documented that flax (C_3_-plant) invested little carbon, but obtained N and phosphorous by up to 94% via CMNs from the sorghum (C_4_-plant) [20], revealing the high dependency of CMN-aided nutrient acquisition from the donor plant. Therefore, these below-ground mycorrhizal networks play important roles in the signal transduction and nutrient sharing between the interconnected plants [21].

Besides the improvement in plant growth and establishment, the AM fungal extraradical mycelium entangles the soil particles and facilitates their aggregation and stabilization [22], thereby improving the soil’s physical properties, such as infiltration rate, water holding capacity, and carbon storage [23,24]. Glomalin-related soil proteins (GRSPs), produced mainly by AM fungi, exhibited substantial functioning in cementing soil aggregates and stabilizing soil structures [18]. It has been reported that GRSPs significantly increased soil stability in the grassland ecosystems of northeast China [25,26]. However, no evidence is available regarding the effect of CMNs on the production of GRSPs and their functioning on soil aggregate stability in the typical steppe.

The typical steppe of Inner Mongolia is the dominant vegetation type in semi-arid areas of northern China [27] and plays an essential role in providing ecological services and life necessities [28,29]. However, anthropogenic activities and climate change have severely degraded steppe grasslands, resulting in decreased soil quality and plant productivity [30,31,32,33]. This grassland system is particularly sensitive to N enrichment because N is a major limiting soil nutrient in this region [34,35] and even a small amount of change in soil N could have significant effects on plant growth and soil quality [36]. Therefore, N fertilization has been extensively used to increase the availability of soil N [37,38] enhance plant production [39,40,41], and improve soil properties [38]. These effects can be boosted by mycorrhizal networks that play an active role in ecosystem functioning and regulate N cycling [42,43]. It has also been observed that increased N availability often results in improved plant productivity but decreases the species diversity of the plants [44,45] and leads to the extinction of susceptible functional groups [46]. Additionally, N enrichment can significantly change the diversity and abundance of soil microbial communities [47,48], causes dormancy, decreases the diversity of the active soil microbial community [49], and weakens the plant–microbe interactions [49]. Global N enrichment is considered to be one of the major threats to the structure and functioning of the ecosystem because of its various negative effects on biotic communities [50]. Therefore, besides the importance of mycorrhizal networks for improving plant growth and soil properties, it is also important to study how the changing environment, such as an increasing amount of terrestrial N deposition, would affect the CMNs’ functioning. Filling this knowledge gap will enable better predictions of the consequence of a change in CMNs functioning under global changing scenarios.

*Cleistogene squarrosa* is a common perennial grass species in the typical steppe of Inner Mongolia. Due to its dominance in various grassland systems, the importance of *C. squarrosa* has been recognized for the development of a sustainable grassland system. Moreover, mycorrhizal networks play a significant role in stabilizing the long-term dominance of plant species in an ecosystem [51]. Therefore, it is important to find the existence of CMNs in the typical steppe of Inner Mongolia and their importance for the growth and development of *C. squarrosa* and its neighboring plants. This research was designed to examine the existence of CMNs between different individuals of *C. squarrosa* species, and to evaluate the functioning of CMNs across an N gradient. We hypothesize that CMNs exist between individual plants of the same species and, if so, we further address the following key questions: How could CMNs affect the plant growth and soil properties of the neighboring plants? How would the functioning of CMNs change under different levels of N?

## 2. Materials and Methods

### 2.1. Experimental Design

**Figure 1:**
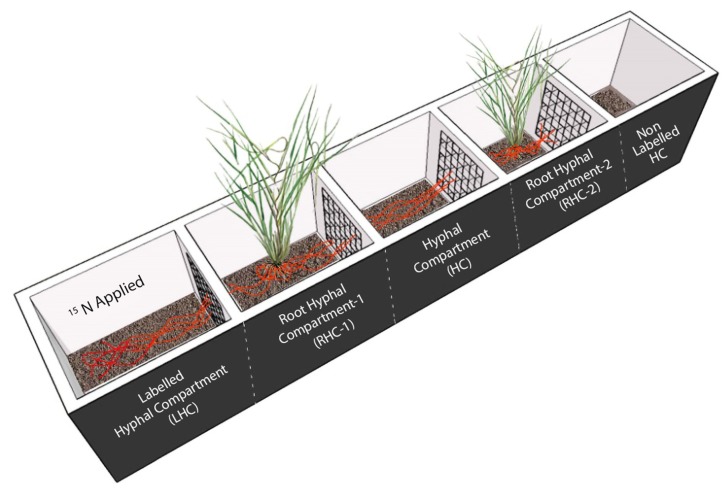
Schematic diagram of five-compartment root-box to grow *C. squarrosa* seedlings. The plant receiving AM fungal inoculum was designated as the donor plant, while the plant without inoculum was the recipient plant. The compartments were separated from each other via 37 µm mesh that only allowed the hyphae to pass, but not the roots. The black lines with the plant show the roots, while red lines show the common mycorrhizal network.

Partitioned root-boxes were constructed and *C. squarrosa* plants were grown in separate compartments to test the existence and functioning of CMNs between two plant individuals under four different N levels. As shown in Figure 1, the root-box was composed of five compartments, each 5 cm long, 5 cm wide and 12 cm high. Two adjacent compartments were separated by a nylon mesh of 37 µm to restrict the root passage but allow mycorrhizal hyphae go through. From left to right, the first compartment was the labeled hyphal compartment (LHC) where ^15^N was applied, the second and the fourth compartments were two root hyphal compartments (RHC1 and RHC2), and in each a fifteen-day old pregerminated seedling of *C. squarrosa* was transplanted, the third compartment was a hyphal compartment (HC) because hyphae from RHC1 could extend into this space, and the fifth compartment was designated as a non-labeled hyphal compartment (NLHC).

The experiment consisted of two AM fungal treatments (mycorrhizal treatment and non-mycorrhizal controls) and four levels of N addition (0, 7.06, 14.15, and 21.19 mg/kg, designated as N0, N1, N2, and N3, respectively), being fully crossed, and each treatment combination being replicated three times, and therefore resulting in a total of 24 root-boxes. For mycorrhizal treatment, the donor plant in the RHC1 compartment received AM fungal inoculum, while the recipient plant in the RHC2 compartment received no AM fungal propagules. For non-mycorrhizal controls, no AM fungal inoculum was added in either RHC1 or RHC2.

### 2.2. Soil, Inoculum and Planting

The soil was collected from a typical steppe of Inner Mongolia (43°38′55.9″N, 116°09′06.3″E) and mixed in an equal proportion with sand (1:1, *v*/*v*). It was then sieved with 2 mm mesh and sterilized with two cycles of the autoclave at 121 °C, 0.11 Mpa for 2 h. Each compartment of the experimental equipment was filled with 1 kg of sterilized soil. The mycorrhizal fungal inoculum was the soil collected randomly from root zones of three native grass species, *C. squarrosa*, *Leymus chinensis*, and *Stipa grandis,* because these plant species showed low host specificity [5]. Collected soil samples were bulked, mixed and stored at 4 °C for AM fungal inoculation. Such soil inoculum consists of soil, the dried root fragments, AM fungal spores, and hyphae, and other microorganisms.

The seeds of *C. squarrosa* were surface sterilized with 70% ethanol for 45 s and washed with distilled water. The seeds were sown on Petri dishes, and four weeks old seedlings were transplanted into the root-box. The plant with 70 g of soil containing AM fungal propagules transplanted into the RHC1 compartment was designated as a donor plant, while the plant in the RHC2 compartment receiving no AM fungal inoculum was called a recipient plant.

To eliminate the effect of non-AM microorganisms, all root-boxes of mycorrhizal and non-mycorrhizal treatments were treated with 15 mL non-sterile soil sievate/microbial wash to include the effect of soil microorganisms other than AM fungi because, during the autoclave of soil samples, biotic factors were eliminated. In this, non-sterilized soil samples were mixed with water to make a soil solution, which was sieved through 38 µm mesh so that AM fungi were suspended on the sieve and other microorganisms were passed through 38 µm mesh with the solution. Then, the obtained solution of the microbial wash was applied to include the effect of other microorganisms, so that the actual effect of AM fungi could be assessed.

All the root-boxes were placed in growth chambers with day/night temperature 24/18 °C, at Beijing Forestry University, Beijing, China. The positioning of root-boxes was changed every week. Plants were grown for 16 weeks in growth chambers.

### 2.3. Labeling with ^15^N

^15^N labeling was performed after 16 weeks of transplantation for 48 h just before the harvesting. For each sample, 1.2 mg of ^15^N was applied in the form of solution (1 mL of 1.2 mg/mL) by dissolving ^15^NH_4_Cl in deionized water with DMPP (3,4-dimethyl pyrazole phosphate) that inhibits the transformation of NH_4_^+^ into NO_3_^−^ [52] and, in the control, an equivalent amount of deionized water was applied instead of ^15^N labeling. The ^15^NH_4_Cl solution was injected with a syringe into 4 cm depth of soil at the center of each chamber of LHC. After that, no further water was applied to the plants.

### 2.4. Analytical Procedures

#### 2.4.1. Plant Harvest

After 48 h of ^15^N labeling, the donor and recipient plants were harvested from both mycorrhizal and non-mycorrhizal treatment. The roots were divided into two parts, one used for the determination of mycorrhizal colonization and the remaining used for the determination of ^15^N. The shoot and root samples were oven-dried at 70 °C for 72 h to measure dry shoot weight (DSW) and dry root weight (DRW). The soil samples collected from the RHC1, HC, and RHC2 were also stored separately at 4 °C for different analyses, like the determination of soil hyphal length density, glomalin-related soil proteins, and soil organic carbon content.

#### 2.4.2. Mycorrhizal Colonization, Hyphal Length Density, and Glomalin-Related Soil Proteins

About 100 root segments (each of 1 cm long) per seedling were cut and cleaned in 10% KOH solution at 90 °C for 10 min and in 2% HCl solution for 10–15 min and stained with trypan blue (0.05%). Finally, the percentage of mycorrhizal colonization was determined by using the magnified intersection method at 200× magnification (Nikon-E100) [53], while, for hyphal length density (HLD), a 5 g soil sample was blended with 50 mL of sodium hexametaphosphate. The supernatant was filtered through a 0.45 µm microporous Millipore membrane by vacuum filtration. The hyphae extracted on each filter paper were stained with 0.05% trypan blue. Finally, the hyphal length density was determined by the grid-line intercept method at 200× magnification and expressed in mg^-1^ [54].

Glomalin-related soil proteins (GRSPs) were extracted according to Wright and Upadhyaya protocol [55]. For easily extractable glomalin-related soil protein (EE-GRSP), 1 g of air-dried soil sample was autoclaved with 8 mL of 20 mM sodium citrate (pH = 7) for 30 min at 121 °C. It was then centrifuged at 5000× *g* for 15 min, and the supernatant was extracted and stored at 4 °C, while, for total extractable glomalin-related soil protein (T-GRSP), 8 mL of 50 mM sodium citrate (pH = 8) was autoclaved for 60 min at 121 °C. The supernatant obtained was stored at 4 °C. Finally, the protein contents in EE-GRSP and T-GRSP supernatant were determined by using the Bradford assay with bovine serum albumin (BSA) as a standard [56].

#### 2.4.3. Chlorophyll Content

The chlorophyll content was measured as described by Ali [57]. Briefly, 100 mg fresh leaf samples were ground with 8 mL of 80% acetone and centrifuged at 4000 rpm for 10 min. Finally, the supernatant was separated, and the OD of the sample was measured at 645, 663, and 470 nm by using UV-spectrophotometer. The equations for the determination of chlorophyll-a, chlorophyll-b, and carotenoids are as follows

Chlorophyll a= 1.07 (OD 663) − 0.09 (OD 645)(1)

Chlorophyll b= 1.77 (OD 645) − 0.28 (OD 663)(2)

Carotenoids= OD 470 × 4(3)

#### 2.4.4. Determination of ^15^N Content

The oven-dried root and shoot samples were milled to a fine powder and sent to the Huake Precision Stable Isotope Laboratory (Shenzhen, China) to detect ^15^N content. ^15^N abundance was found by using an elemental analyzer coupled with isotope ratio mass spectrometer (Thermo Fisher Scientific Inc., Waltham, MA, USA). The ^15^N content in tissues (shoot and root) were calculated as the product of the tissue dry biomass and its ^15^N concentration (^15^N content = Biomass × ^15^N concentration). By subtracting the average total of ^15^N content in control from the total ^15^N content in each treatment, we got the net ^15^N content for each treatment. To calculate the hyphal N contribution, the net ^15^N content from the recipient plant was divided by the net ^15^N content from the donor and recipient plants uptake by hyphae, and to show in % age, multiplied by 100.

(4) $$N\;\mathrm{transfer}\;(\%)=\frac{{}^{15}N\;\mathrm{in}\;\mathrm{recipient}\;\mathrm{plant}}{{}^{15}N\;\mathrm{in}\;\mathrm{donor}\;\mathrm{plant}\;+{}^{15}N\;\mathrm{in}\;\mathrm{recipient}\;\mathrm{plant}}\;\times 100$$

#### 2.4.5. Water-Stable Aggregates, Mean Weight Diameter and Soil Organic Carbon

The water-stable aggregates (WSA) were measured according to the wet sieving method [58]. In short, a series of three sieves were used to collect the four aggregate size fractions: (a) 2–1 mm, (b) 1–0.5 mm, (c) 0.5–0.25 mm, (d) <0.25 mm. A total of 5 g air-dried soil samples were pre-wetted by submerging in distilled water at room temperature for 30 min to equilibrate. The aggregate fractions were separated manually by moving the sieves up and down by up to 3 cm in water for 2 min with 50 repetitions. After that, the aggregate fractions on each sieve were collected and oven-dried at 65 °C for 48 h until a constant weight was achieved. The WSA fractions were expressed as the percentage of WSA against the total dry soil sample. WSA stability was calculated in terms of MWD of stable aggregates, as given below [59];

(5) $$\mathrm{MWD}\;\left(\%\right)=\overset{n}{\underset{i=1}{\sum }}XiWi$$

where Xi denotes the diameter of sieve (mm), Wi shows the proportion of size fractions in the total sample weight, and n is the number size fractions (*n* = 4).

Soil organic carbon (SOC) content (g kg^−1^) was measured according to the dichromate oxidation method [60].

#### 2.4.6. Statistical Analysis

Two-way analysis of variance was performed to analyze the effect of two factors (mycorrhizal treatments and N addition). A *t*-test was performed to specifically check whether non-mycorrhizal controls differed from the mycorrhizal treatments. We performed the least significant difference (LSD_0.05_) and Tukey test to analyze the difference between the treatments (Statistix 8, version 8.1). Correlation analyses between the two variables were assessed using the Spearman correlation by using SPSS 25.0 software package.

## 3. Results

### 3.1. AM Fungal Colonization and Mycorrhizal Network

**Figure 2:**
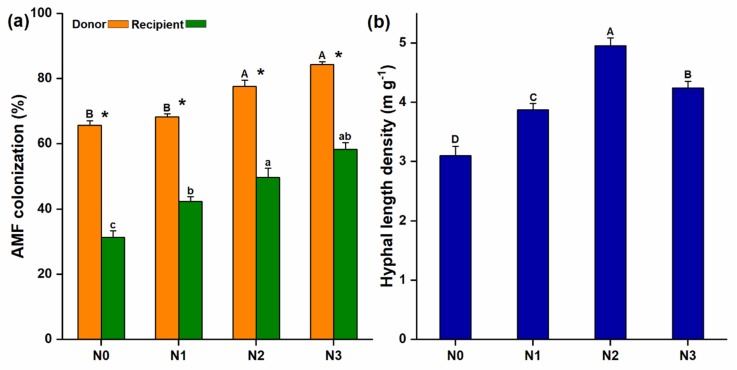
Arbuscular mycorrhizal (AM) fungi colonization and the development of common mycorrhizal network; (**a**) AM fungal colonization was observed in both donor and recipient plant; (**b**) After inoculation, extraradical hyphae were developed, which were measured from the hyphal compartment; the same lowercase and uppercase letters indicate non-significant differences among different N treatments of the respective donor and the recipient plants, while (*), shows a significant difference between donor and recipient plants at different N-treatments.

Microscopic examination revealed the presence of AM fungal extraradical hyphae extending from the donor to the recipient compartment through the 37 µm mesh (Figure S1a) and the recipient plant roots were also colonized by the AM fungi (Figure S1b). The mycorrhizal colonization of the donor plants was considerably higher than that of the recipient plants and significantly increased with increasing soil N levels (Figure 2a). AM fungal hyphal length density (HLD) also varied under different levels of N additions and significantly higher HLD was observed at N2 (4.96 ± 0.08 m g^−1^) (Figure 2b).

### 3.2. Plant Biomass and Chlorophyll Content

The inoculation of the donor plant with AM fungi increased the plant biomass of both the donor and the recipient plant as compared to the non-mycorrhizal plants. The average dry shoot weight (DSW) and dry root weight (DRW) were not significantly different between donor and recipient plants in the non-mycorrhizal treatment. However, in mycorrhizal treatment where the donor plant was inoculated with AM fungi, the average DSW (Figure S2a) and average DRW (Figure S2b) of the donor and recipient plants was increased as compared to non-mycorrhizal treatment. Hence, the maximum average DSWs observed at N2 for the donor and recipient plants were 3.60 and 2.86 g, respectively. Similarly, DRW was also found at a maximum at N2 for the donor (1.48 g) and recipient (1.18 g) plants. A significant interaction between AM fungal inoculation and N treatment was found for DRW in both donor and recipient plants, while for DSW, only in the recipient plant was a significant interaction effect found (Table 1).

CMNs increased the chlorophyll content (Chl-a, Chl-b, and carotenoids) in the donor and recipient plants in the mycorrhizal treatment as compared to non-mycorrhizal treatment (Figure S3). For mycorrhizal treatment, chlorophyll content in the donor plants increased significantly compared to that in the recipient plants at a different N-treatment, while no significant difference was found in chlorophyll content between the donor and recipient plants of non-mycorrhizal treatment. The mycorrhizal and non-mycorrhizal treatments showed the highest chlorophyll content at N3, and lowest at N0, treatment. AM fungal inoculation and N treatment had significant interaction effects on Chl-b and carotenoids contents (Table 1).

### 3.3. ^15^N Transfer

**Figure 3:**
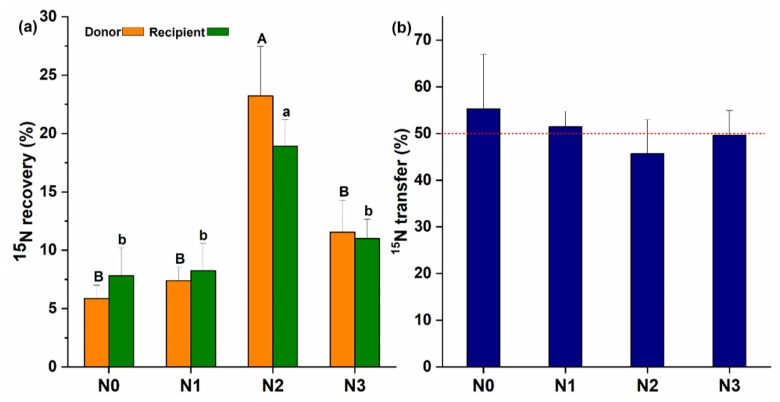
Role of common mycorrhizal networks (CMNs) in the recovery and transfer of ^15^N; (**a**) the amount of ^15^N uptake by mycelium and recovered in donor and recipient plants; (**b**) % of N^15^ transfer from donor to recipient plant with help of the mycorrhizal networks. Capital letters show the difference between donor, and small letters are showing differences among recipient plants at different treatments (N0, N1, N2, and N3). Alphabetic on the top of each bar shows the LSD_0.05_ difference.

The establishment of CMNs between the inoculated donor and non-inoculated recipient plant was confirmed with the transfer of ^15^N from the donor to the recipient plants. The highest ^15^N content was recovered at N2 treatment in the donor and recipient plants (Figure 3a) as compared to other N treatments. ^15^N content in the donor plants was 5.86%, 7.40%, 23.22%, and 11.55% for N0, N1, N2, and N3, respectively, and the difference was significant (F (3, 8) = 8.77, *p* = 0.006). The ^15^N content recovered in the recipient plants also showed a significant difference for different N-treatments (F (3, 8) = 5.60, *p* = 0.023) with the average being 7.82%, 8.24%, 18.93%, and 11% for N0, N1, N2 and N3, respectively. Moreover, CMNs’ transfer rates of ^15^N from the donor to the recipient plants were about 55.31%, 51.44%, 45.65%, and 49.59% for N0, N1, N2 and N3, respectively, and non-significant differences were found for the transfer of ^15^N content from the donor to recipient plant at different N levels (F (3, 8) = 0.28, *p* = 0.837) (Figure 3b). In the non-mycorrhizal treatment, no AM fungal colonization or extraradical hyphae was observed and no ^15^N (%) transfer was recorded.

### 3.4. Soil Properties

In the mycorrhizal treatment, easily extractable glomalin-related soil protein (EE-GRSP; Figure S4a) and total glomalin-related soil protein (T-GRSP; Figure S4b) were higher than the non-mycorrhizal treatment. In the mycorrhizal treatment, the production of EE-GRSP under the donor and recipient plants peaked at N2, averaging about 0.137 and 0.129 mg/g, respectively. T-GRSP content was also the highest at N2 treatment for the donor (0.432 mg/g) and the recipient (0.410 mg/g) plants. Significant differences were found between the donor and recipient plants in mycorrhizal treatments, while non-significant differences were observed for non-mycorrhizal (Figure S4). There was a significant interaction effect of AM fungal inoculation and N treatment on both GRSP fractions (Table 1). Significant differences were found for EE-GRSP and T-GRSP for different N-treatments.

In the mycorrhizal treatment, the percentage of water-stable aggregates (WSA) at the size of 2–1, 1–0.5, 0.5–0.25 mm was increased as compared to the non-mycorrhizal treatment (Table S1). Mean weight diameter (MWD) was higher in the mycorrhizal treatment as compared to the non-mycorrhizal treatment. (Figure S5). A significant interaction between AM fungal inoculation and N treatment occurred for MWD (Table 1).

Compared with the non-mycorrhizal treatment, the mycorrhizal treatment increased SOC by 46%, 27%, 15% and 23% in the donor plant and 35%, 18%, 03%, and 18% in the recipient plant, respectively (Figure S6). A significant interaction was found between AM fungal inoculation and N treatment for SOC (Table 1).

### 3.5. Relationship Between AM Fungi and Soil Properties

**Figure 4:**
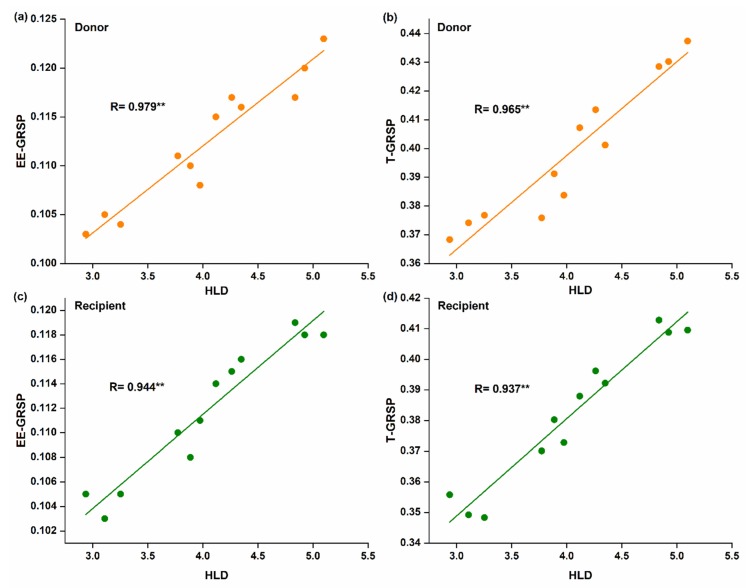
Linear correlation between hyphal length density and glomalin-related soil proteins (GRSPs) fractions; (**a**,**b**) in the donor plant; (**c**,**d**) in the recipient plants (*n* = 12).

Spearman correlation analysis showed that hyphal length was positively and significantly correlated with EE-GRSP (*R* = 0.979 for donor, Figure 4a; and *R* = 0.944 for recipient plant, Figure 4c) and T-GRSP (*R* = 0.965 for donor, Figure 4b; and *R* = 0.937 for recipient plant, Figure 4d).

**Figure 5:**
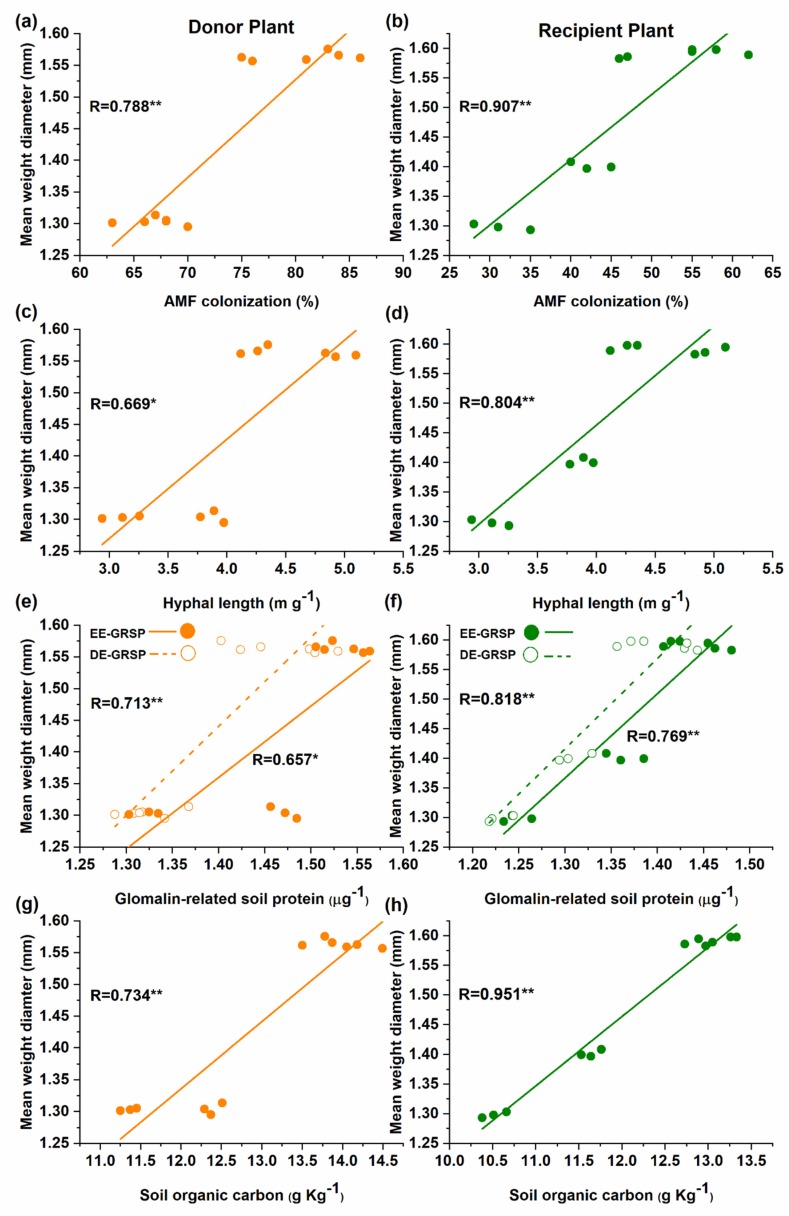
Linear correlation in the donor and recipient plants between mean weight diameter (MWD) and; (**a**,**b**) colonization or; (**c**,**d**) hyphal length or; (**e**,**f**) glomalin-related soil proteins (GRSPs) fractions or; (**g**,**h**) soil organic carbon (SOC) in mycorrhizal treatment (n = 12).

The correlation further verified the significant positive relationship between the mean weight diameter (MWD) with AM fungal colonization (Figure 5a,b), hyphal length (Figure 5c,d), EE-GRSP and T-GRSP (Figure 5e,f), SOC (Figure 5g,h) in both donor and recipient plants in the mycorrhizal treatment.

## 4. Discussion

In this study, the mycorrhizal network was established between the inoculated donor and non-inoculated recipient plants of *C. squarrosa*. Inoculation in the donor plant resulted in root AM fungal colonization and, as a result, the extraradical hyphae from a donor plant moved to the recipient plant and formed AM fungal colonization. This AM fungal colonization in the recipient plant led to the formation of hyphal connection between the donor and recipient plant, called a common mycorrhizal network. This was consistent with the results of [61], who found that the inoculation of a donor plant with AM fungi resulted in the formation of the mycorrhizal network between trifoliate orange and white clover.

Different levels of N fertilization were applied to assess the efficiency of AM fungal colonization and the mycorrhizal network to observe the transfer of nutrients from the donor to recipient plants and their effects on the neighboring plants. It was found that N fertilization had a significant effect on AM fungal colonization and soil hyphal length, which is in line with the previous findings that N-fertilization plays an important role in increasing the AM fungal colonization [62]. As a result, plant shoot weight, root weight and chlorophyll content in the donor and recipient plants increased as compared to non-mycorrhizal plants. These findings are in agreement with previous studies of trifoliate orange-white clover and flax-sorghum, and *Andropogon gerardii*, where plant biomass was improved as a result of mycorrhizal networks [20,57,61,63,64,65]. This is because these extraradical radical hyphae provided the increased nutrients absorption surface for the plants. The maximum ^15^N recovery was found at N2 treatment in the donor and recipient plants, suggesting that the recovery of ^15^N decreased at a high level of N fertilization [66]. Thus, mycorrhizal plants used the nutrients more effectively than non-mycorrhizal plants, resulting in increased plant biomass at N2 treatment. This increase in plant biomass provided a larger sink for ^15^N recovery at N2. Therefore, as a result, a lower transfer rate was found at N2 treatment as compared to other N-treatments. Another reason could be that the recovered ^15^N at N2 was efficiently utilized by the donor plant to produce the higher plant biomass, and hence found a lower transfer rate at N2, which was also reported previously [66]. Therefore, AM fungal inoculation and CMNs formation increase plant biomass [57,63]. This effect was also observed in maize plants [62]. We found that CMNs in *C. squarrosa–C. squarrosa* association significantly increased the root and shoot weight of the recipient plant, suggesting that CMNs can enhance the plant growth performance of the neighboring plants. The other reason for the increase in plant biomass was due to an increase in chlorophyll content at high N level. However, the mycorrhizal plants showed higher chlorophyll content as compared to the non-mycorrhizal plants. The higher chlorophyll content might be due to more chloroplasts existing in the bundle sheath of inoculated plants [67]. It was also observed the AM fungal inoculation increased the chlorophyll content in the plants [67,68]. Moreover, other reasons might be the increased stomatal conductance, higher photosynthesis and transpiration rate, and improved plant growth. Therefore, in the mycorrhizal plants, significant effects of N-fertilization were found, with increased plant biomass and chlorophyll content.

The mycorrhizal network significantly improved plant growth by the redistribution of nutrients to their neighboring plants. In this study, the mycorrhizal network transferred soil N from the donor to recipient plants on an average of 50%. The ability of the mycorrhizal network to transfer N from the donor to recipient plant varies from 0–80% [69]. However, much elevated N-fertilization did not increase nutrient transfer and plant biomass because plants may face other limitations, like water, light or space, that limit their growth [70]. However, at severe N deficiency, the AM fungi might consume additional N sources for their own needs, and as a result, there is little or no N transfer to the plants [71]. It is also observed that the amount of N transfer is correlated with mycorrhizal colonization level and hyphal length density [72]. Moreover, it is well documented that the fungal partner makes a significant contribution to the uptake in soil nutrients mediated by the mycelial network [73,74,75,76], making a significant contribution to improve the performance of the neighboring plants.

Soil aggregability was improved with the help of CMNs, as the aggregation of soil particles of different sizes stabilizes the soil organic carbon [77]. Soil aggregation may be influenced by various factors, such as soil biota, clay, and soil organic carbon [78]. It is observed that AM fungi can play a significant role in the stabilization of soil particles by the GRSP contents released by the mycelial network. [18]. In this study, the average EE-GRSP and T-GRSP contents were higher in mycorrhizal treatment than non-mycorrhizal, which is consistent with the previous finding [79]. The GRSPs’ contents increased with the addition of N, and the maximum GRSP fractions were found at N2. However, after a certain increase in N, the GRSP contents decreased, and similar findings were observed by Sun et al. (2018). This is because, in the beginning, the addition of N quickly relieves the N deficiency in the soil, thereby encouraging the microbial activities to stimulate the production of GRSPs. However, after a certain increase of N results in N saturation into the soil and inhibits microbial activities, the production of GRSPs would decrease [80]. Therefore, N addition has the potential to increase the GRSPs in the soil [61,81]. AM fungal inoculation played a significant role in the production of GRSPs in both the donor and recipient plants. Moreover, a significant positive correlation was found with soil hyphal length (Figure 4), as reported previously [61,82], which improved the soil structure. These findings are supported by researches showing that the percentage of water-stable aggregates increases with the increase in hyphal length in the pot experiments [61,82,83]. Moreover, the long term field experiments in grassland ecosystems also found a positive correlation of hyphal length with water-stable aggregates [83]. In our case, we also found that the percentage of water-stable aggregates (WSA) at sizes of 2.00–1.00 and 1.00–0.50 mm was significantly higher in the mycorrhizal treatment as compared to non-mycorrhizal treatment (Table S1). Thus, the mycorrhizal hyphae entangle the soil particles and stabilize the macroaggregates [84] and the GRSPs help in the binding of these macroaggregates [85].

Besides the GRSPs, it has been widely accepted that AM fungi also play a key role in soil carbon storage, by either depositing organic compounds such as chitin and glomalin in the rhizosphere [26,86] or protecting the soil organic matter from the microbial decomposition through promoting aggregate stability [87]. Mycorrhizal plants have more SOC contents than non-mycorrhizal plants in our study, which supports previous findings [79]. Our findings also revealed that MWD significantly increased in the mycorrhizal treatments, and a significant positive correlation of MWD was found with AM fungal colonization, hyphal length, EE-GRSP, T-GRSP, and SOC (Figure 5). This implies that GRSPs fractions, AM fungal colonization, hyphal length, and SOC all significantly improved the soil aggregate stability [82,88,89]. For example, the addition of N can increase the AM fungal colonization with a low N level [90], thereby increasing the GRSPs’ fractions in the soil [91]. However, N addition beyond a certain level decreases the GRSPs in the soil [92]. Interestingly, the addition of N significantly increased the SOC in the soil of mycorrhizal treatment as compared to non-mycorrhizal treatment [93]. SOC is considered an important component of soil fertility and therefore AM fungal inoculation and the subsequent formation of CMNs enhance the soil fertility and improve the growth of donor and recipient plants. This suggests that a common mycorrhizal network would persuade the well-developed mycelium and GRSP fractions (EE-GRSP and T-GRSP), and SOC in the rhizosphere of the recipient plant, resulting in the improvement of soil aggregate stability and the growth of the recipient plant.

## 5. Conclusions

This study revealed for the first time that CMNs exist between individuals of *C. squarrosa* in a constrained environment. Colonization of AM fungi occurred in the donor roots and subsequent CMN formation caused root colonization in the recipient plant. AM fungal inoculation increased the plant biomass, chlorophyll content, and EE-GRSP, T-GRSP, MWD, and SOC. Moreover, the CMNs originating from the donor plant also facilitated and improved plant growth and soil properties in the recipient plant. These findings suggest that AM fungal inoculation and the subsequent establishment of CMNs can play important roles in improving soil aggregation, soil fertility and plant growth. Therefore, this study confirms the existence of mycorrhizal networks in the typical steppe of Inner Mongolia. This provides a basis for understanding the mechanism of intra-plant communication in association with plant growth and development in this grassland system.

## Figures and Tables

**Table 1:** Mean squares of absolute values for various traits under mycorrhizal and non-mycorrhizal treatment and significance of main treatment effects (N and Fungus) and their interaction effects (N × Fungus) based on two-way ANOVA.

Parameters	Source of Variation	Nitrogen	Fungus	Nitrogen × Fungus	Residual
	df	3	1	3	14
Dry shoot weight	Donor	1.50^**^	28.93^**^	0.01^ns^	0.02
	Recipient	1.17^**^	14.09^**^	0.05^*^	0.01
Dry root weight	Donor	0.99^**^	0.95^**^	0.05^**^	0.00
	Recipient	0.77^**^	0.29^**^	0.02^**^	0.00
Chlorophyll-a	Donor	0.52^**^	0.62^**^	0.00^ns^	0.00
	Recipient	0.51^**^	0.18^**^	0.00^ns^	0.00
Chlorophyll-b	Donor	2.03^**^	0.57^**^	0.01^*^	0.00
	Recipient	2.01^**^	0.26^**^	0.01^**^	0.00
Carotenoids	Donor	41.34^**^	81.60^**^	2.71^**^	0.02
	Recipient	33.44^**^	19.03^**^	0.96^**^	0.01
EE-GRSP	Donor	0.00^**^	0.00^**^	0.00^**^	0.00
	Recipient	0.00^**^	0.00^**^	0.00^**^	0.00
T-GRSP	Donor	0.00^**^	0.17^**^	0.00^**^	0.00
	Recipient	0.01^**^	0.14^**^	0.00^**^	0.00
SOC	Donor	15.98^**^	41.92^**^	0.73^**^	0.04
	Recipient	15.52^**^	17.58^**^	1.54^**^	0.04
MWD	Donor	0.07^**^	0.56^**^	0.01^**^	0.00
	Recipient	0.07^ns^	0.71^**^	0.01^**^	0.00

*, **and ns indicate the significant differences at *p* ≤ 0.05, *p* ≤ 0.01, and non-significant (*p* ≥ 0.05), respectively.

## Supplementary Materials

Supplementary materials can be found at https://www.mdpi.com/2076-2607/8/2/230/s1.
