# Supplementary material for: Formation of Common Mycorrhizal Networks Significantly Affects Plant Biomass and Soil Properties of the Neighboring Plants under Various Nitrogen Levels
Source: Microorganisms. 2020 Feb 8;8(2):230. doi: 10.3390/microorganisms8020230 (PMC7074789; doi:10.3390/microorganisms8020230)
Supplement: Supplementary file 1 [file microorganisms-08-00230-s001.pdf]

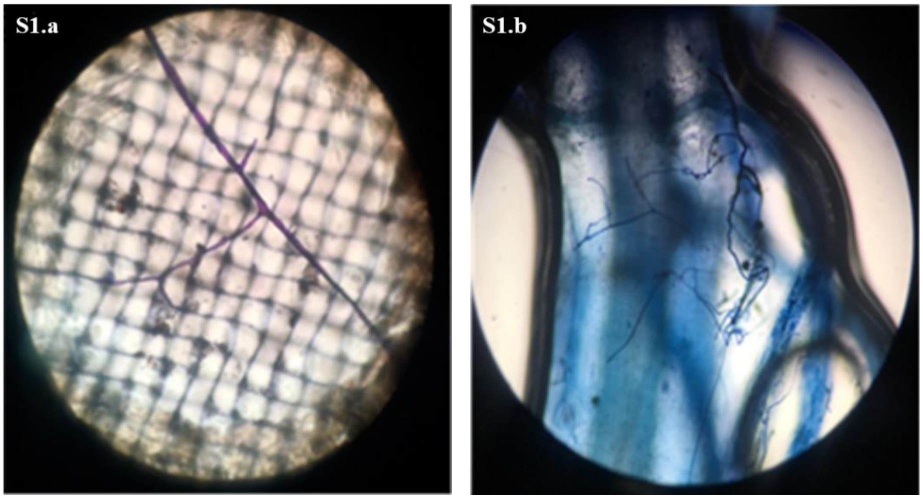

Figure S1. (**a**) AM fungal extraradical hyphae crossing through the mesh of the recipient compartment; **(b)** AM fungal colonization was observed in the recipient compartment at 200× magnification.

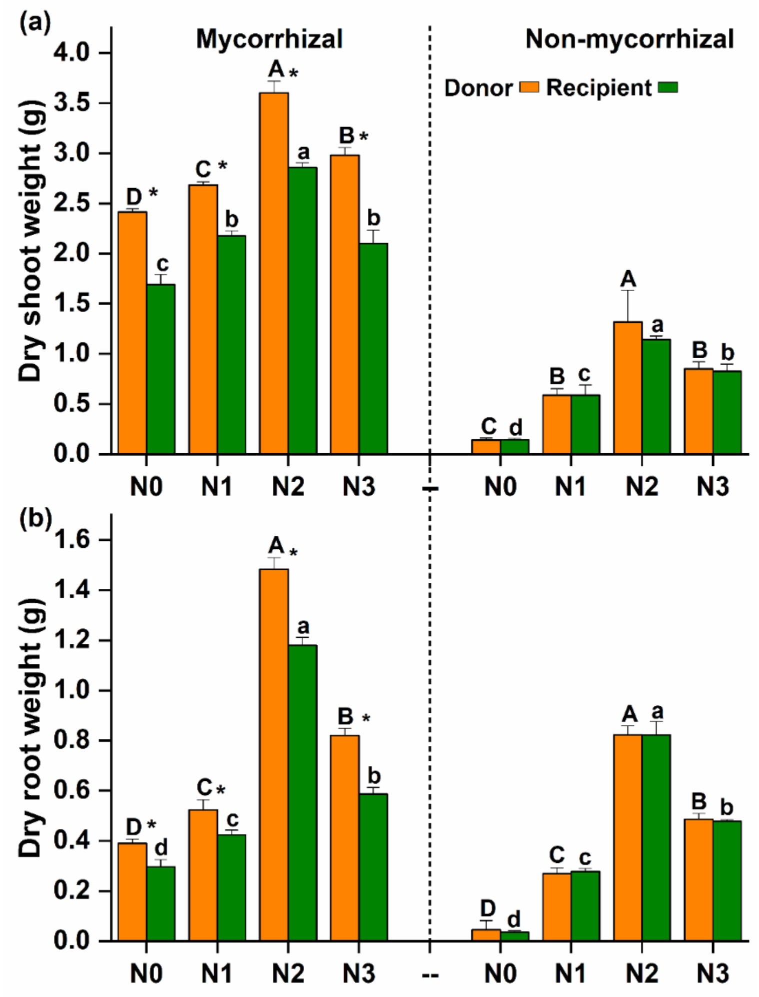

Figure S2. A gain in plant biomass of shoot and root of *C. squarrosa* seedlings. The inoculation with AM fungi increased shoot and root weight in mycorrhizal treatment as compared to non-mycorrhizal treatment; (**a**) dry shoot weight (DSW); (**b**) dry root weight (DRW); Alphabetic on the top of each bar is showing the $LSD_{0.05}$ difference. Capital letters are showing the difference between donor, and small alphabets are showing difference among recipient plants at different treatments (N0, N1, N2, and N3). The symbol * is showing the significant difference between donor and recipient plants at different N-treatment.

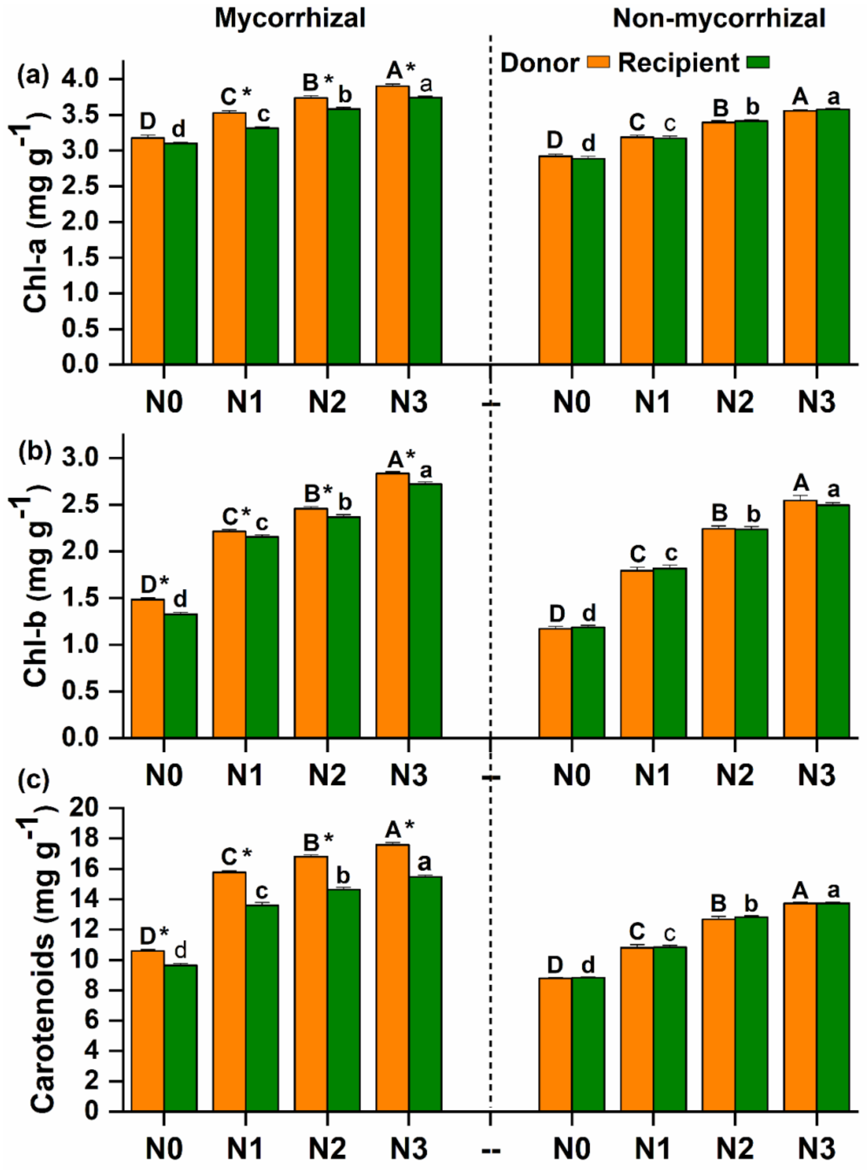

Figure S3. Chlorophyll content in *C. squarrosa* seedlings. The inoculation with AM fungi increased the chlorophyll content in mycorrhizal treatment as compared to non-mycorrhizal treatment; (**a**) Chl-a; (**b**) chl-b; (**c**) carotenoids. Alphabetic on the top of each bar is showing the $LSD_{0.05}$ difference. Capital letters are showing the difference between donor, and small alphabets are showing differences among recipient plants at different treatments (N0, N1, N2, and N3). The symbol * is showing the significant difference between donor and recipient plants at different N-treatment.

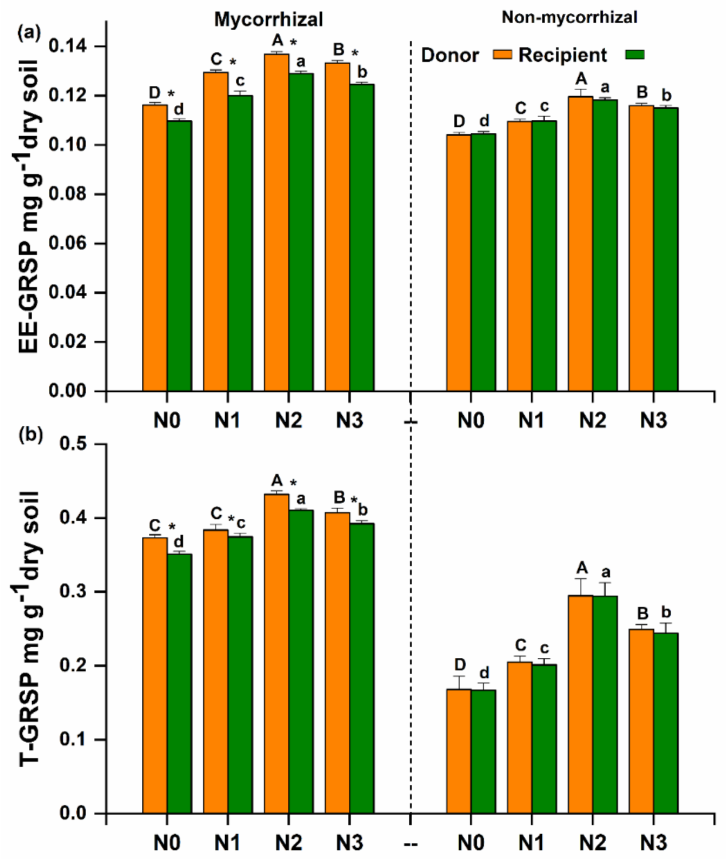

Figure S4. Soil concentration of glomalin-related soil proteins (GRSPs). Production of; (**a**) EE-GRSPs; (**b**) T-GRSPs in mycorrhizal and non-mycorrhizal treatments. Alphabetic on the top of each bar is showing the $LSD_{0.05}$ difference. Capital letters are showing the difference between donor, and small alphabets are showing difference among recipient plants at different treatments (N0, N1, N2, and N3). The symbol * is showing the significant difference between donor and recipient plants at different N-treatment.

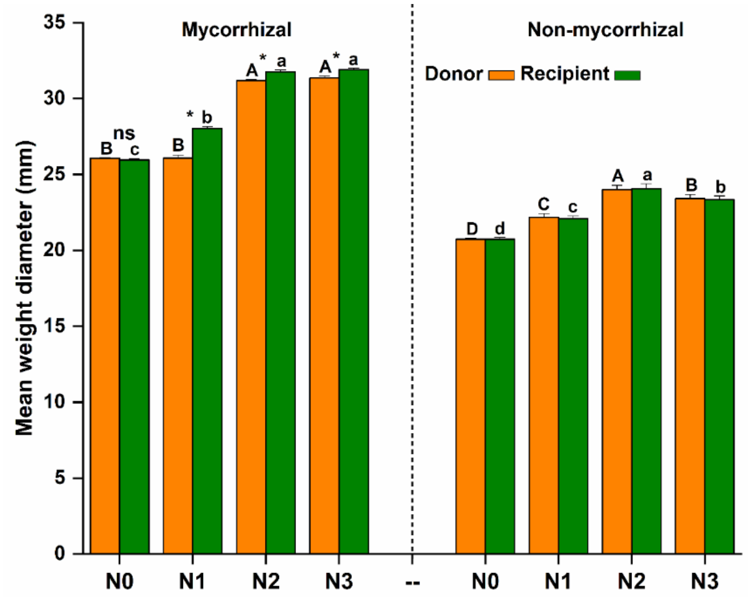

Figure S5. Mean weight diameter (MWD). Each value represents the mean ± SD (*n* = 3) of mycorrhizal treatment and non-mycorrhizal treatment. Different letters show the statistically significant differences between different nitrogen treatments. Alphabetic on the top of each bar is showing the $LSD_{0.05}$ difference. Capital letters indicate the difference between donor, and small letters are showing difference among recipient plants at different nitrogen treatments. The symbol * shows the significant difference between donor and recipient plants for MWD at each nitrogen treatment.

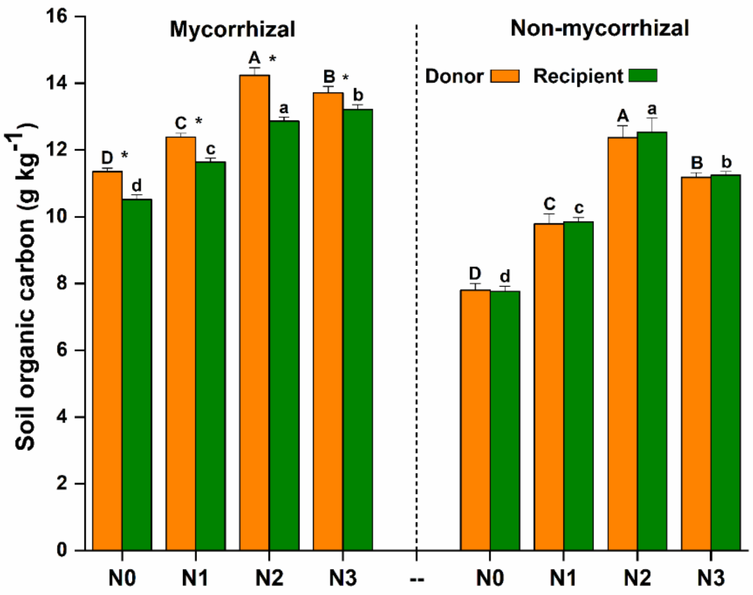

Figure S6. Increase in soil organic carbon (SOC). Each value represents the mean ± SD (*n* = 3). Different letters show the statistically significant differences between different nitrogen treatments. Alphabetic on the top of each bar is showing the $LSD_{0.05}$ difference. Capital letters indicate the difference between donor, and small alphabets are showing differences among recipient plants at different nitrogen treatment. The symbol * is showing the significant difference between donor and recipient plants at different N-treatment.

Table S1. Water-stable aggregates distribution in mycorrhizal and non-mycorrhizal treatment at different levels of nitrogen.

	Treatments	N0		N1		N2		N3	
	**Size**	**Donor**	**Recipient**	**Donor**	**Recipient**	**Donor**	**Recipient**	**Donor**	**Recipient**
**Mycorrhizal**	2-1mm	1.020±0.092C	1.020±0.060d	0.847±0.103D	2.153±0.091c	3.887±0.050B	2.300±0.080b	4.453±0.090A	4.286±0.061a
	1-0.5mm	4.986±.110C	4.513±0.061c	4.513±0.050D	5.240±0.080b	6.493±0.071A	6.446±0.061a	5.300±0.072B	5.300±0.060b
	0.5-0.25mm	36.193±.130C	36.940±0.092d	38.400±0.600A	37.180±0.060c	37.133±0.416B	48.267±0.090a	37.68±0.111B	40.780±0.092b
	<0.25mm	57.067±0.131A	57.533±0.031a	56.933±0.558B	55.067±0.128b	52.467±0.344C	43.740±0.163d	52.80±0.186C	52.133±0.14c
**Non-mycorrhizal**		**Donor**	**Recipient**	**Donor**	**Recipient**	**Donor**	**Recipient**	**Donor**	**Recipient**
	2-1mm	0.080±0.020D	0.080±0.040c	0.180±0.020C	0.140±0.020c	0.267±0.031B	0.260±0.040b	0.470±0.056A	0.480±0.040a
	1-0.5mm	3.260±0.100D	3.250±0.100d	3.573±0.081C	3.550±0.121c	4.347±0.081A	4.380±0.080a	3.853±0.090B	3.820±0.060b
	0.5-0.25mm	24.367±0.070D	24.367±0.101d	28.787±0.729C	28.693±0.699c	33.673±0.921A	33.833±1.213a	31.400±0.596B	31.173±0.718b
	<0.25mm	72.293±0.162A	72.353±0.170a	67.640±0.817B	62.620±0.676b	61.713±0.998D	61.527±1.229d	64.287±0.726C	64.527±0.778c

Data (means ± SD, n = 3). Different letters (capital letters for donor and small letters for recipient) indicates the significant differences (p< 0.05) between different nitrogen treatments.
